# Incidents of Practice

**Published:** 1903-03

**Authors:** 


### Incidents of Practice.

Oil of Cloves.—W. A. Briggs, M.D., of Sacramento, Cal., reports in *American Medicine*, January 3, 1903, an extended experience with clove oil as a disinfectant for the hands of the surgeon, obstetrician, and nurse, and also for the operative field. He has used it in varying strength, with a diluent of olive or other indifferent oil—from pure to twenty per cent.—as a dressing for lacerated, punctured, incised, and contused wounds, and for the umbilical cord; all with marked success and satisfaction. He sums up its advantages,—viz., 1. Practically non-toxic. 2. Locally distinctly analgesic and powerfully antiseptic. 3. A quick, ready, and simple means of accomplishing results.

Note.—Its valuable properties have perhaps been overlooked, undervalued, or forgotten by many in dental practice.—H.

---

Observation of Case of Resorption reported on Page 633 of the International Dental Journal for 1902.—On presentation, November, 1902, the pulp continued vital with a reaction of —56 + 130. This being in close relation with the normal rate of the sound teeth, namely, —58 + 130, it may be considered that the pulp gives indication of continuance of vitality.

The peculiar condition of the gingiva at the lingual aspect had nearly disappeared.—L. J.

---

Traumatic Impaction of Both Deciduous Centrals.—By the fall of a child of two and a half years, March 3, 1897, the centrals were driven into the adjacent tissues so far that the incisal edges scarcely appeared. Within an hour ether was administered, when the teeth were drawn into position, ligated to each other and to the laterals. Tincture calendula one to four of water was sprayed over the parts and mild disinfection was maintained for two days. The teeth became firmly attached in a short time and maintained indications of having living pulps. This condition was confirmed at the removal on their loosening in December of 1899. The absorption of the roots was complete, leaving the residue of the living pulps in the caps of enamel. The permanent teeth did not appear until October, 1900, at the age of six years.—L. J.

